# Influence of education level and gender of dental students on perception of dental aesthetics

**DOI:** 10.1186/s12903-024-04115-x

**Published:** 2024-03-28

**Authors:** Ines Kovačić, Manuela Miloš, Matej Kurkutović, Asja Čelebić, Nikola Petričević

**Affiliations:** Department of Prosthodontics, School of Dental Medicine, University of Zagreb, Gundulićeva 5, Zagreb, 10000 Croatia

**Keywords:** Targeted education, Knowledge, Assisting, Gender, Smile aesthetics, Photograph modifications

## Abstract

**Background:**

Fulfilling aesthetic norms is an imperative of modern society. Accordingly, aesthetics has found its important role in dental medicine. The aim was to study whether there is a difference in the perception of tooth shade change and anatomical variations of anterior maxillary teeth among dental students depending on the level of education, gender, and experience in assisting in a dental office.

**Methods:**

The research was conducted among dental students via the Google Forms platform. Students evaluated the aesthetic acceptability of a smile on the unaltered, reference photograph (RP) and on the other 28 photographs in which the tooth shade (lighter and darker teeth), length (shortened central incisors, elongated, and beveled lateral incisors, elongated canines) and position (atypical and typical rotations and diastemas) of the upper maxillary teeth were altered by digital manipulation. The 1–10 assessment scale was used. Statistical analysis comprised one-way Kolmogorov-Smirnov test, *X*^2^ tests, t-test, and 3-way MANOVA.

**Results:**

In the research 208 students participated, 113 were preclinical students, 104 assisted in dental office and 175 were females. There were no significant effects of gender regarding length and position modifications (*p* > .05), while women were stricter in evaluation of lighter shade (*p* < .05). Clinical students were stricter in evaluating all manipulated photographs (MP) except those towards darker shade modifications, while assisting in a dental office or having a dentist in close family showed no significant effects (*p* > .05).

**Conclusions:**

With a higher level of targeted academic education, dental students sharpen their ability to notice deviations from the aesthetic norms of a smile.

## Background

A smile has an important role in facial expression. It affects a person’s perception of attractiveness and is the foundation of social interaction [1]. It is believed that more attractive people find it easier to get better jobs, have happier marriages, and have a more fulfilling life [1, 2]. On the contrary, poor dental aesthetics play a major role in the overall impression of attractiveness and is often associated with a lack of self-confidence, which can result in deficiency in an individual’s social, business, and academic performances [2].

Aesthetic norms and references come from a variety of sources: principles of aesthetics in art, average measures of specific ethnic communities, observation of people considered attractive, etc. [3–5]. Also in modern times, the influence of social media plays a big role in people’s perception of esthetics [6, 7]. Although a perfect smile is a subjective impression, it can be defined as: “a smile that has a harmonious correlation of the shape and color of the teeth and a good proportion between the lips and the gingiva” [8]. Machado [9] presented aesthetic guidelines for achieving optimal smile characteristics: “incisal edges of the central maxillary incisors must be below the line of the incisal edges of the upper canines which ensures the dominant appearance of the central incisors”. He also pointed out that diastemas in the aesthetic zone are unacceptable, with exception of diastema up to 0.5 mm between the lateral maxillary incisors and canines, which laypeople have not noticed. His research also reported that the need for symmetry is greater around the midline of the face, while mild asymmetries in the lateral region are more acceptable. Patients with good oral health and high education level are more concerned about their orofacial appearance [10].

Perception of aesthetics has been widely studied among the general population and dentists of various levels of professional education [11–15]. Kokich et al. [11] proved that the degree of education impacted the perception of a smile’s aesthetics since the general population was the least critical in the evaluation. Other researchers reported that minor irregularities in the positioning of the incisal edges of the maxillary central incisors do not affect the perception of smile aesthetics among laypeople while changes are symmetrical, but even a slight asymmetric irregularity of the incisal edge was perceived as unattractive [12–14].

While many studies prove that the perception of aesthetic modification is different between general dentists, dentists with different specializations and layperson, there is not enough data about dental students’ progress in perception of aesthetic modification [16–21]. The purpose of this research was to determine whether there is a difference in the perception of modifications in tooth shade, dimensions, and tooth rotations of the maxillary front teeth among the dental students, regarding gender, degree of education, previous experience in dentistry through assisting in dental practice, and the presence of a dentist in the immediate family.

## Methods

The research was approved by the Ethics Committee of the School of Dental Medicine, University of Zagreb (No. 05-PA-30-XXVI43 / 2021). The research was conducted online using the Google forms platform. Students from the first to the sixth year of the School of Dental Medicine, University of Zagreb participated in the research. Before inclusion, the informed consent was obtained, and students completed the Farnsworth–Munsell 100 Hue Test (X-Rite, Grand Rapids) to test the ability of color discrimination. Those with the error score above 20 were excluded. Participation was voluntary and anonymous.

Based on the data obtained in the previous study [12], the minimum number of participants was set at 95 for each group (alpha = 0.05, power = 80%).

### Reference photograph

**Fig. 1:**
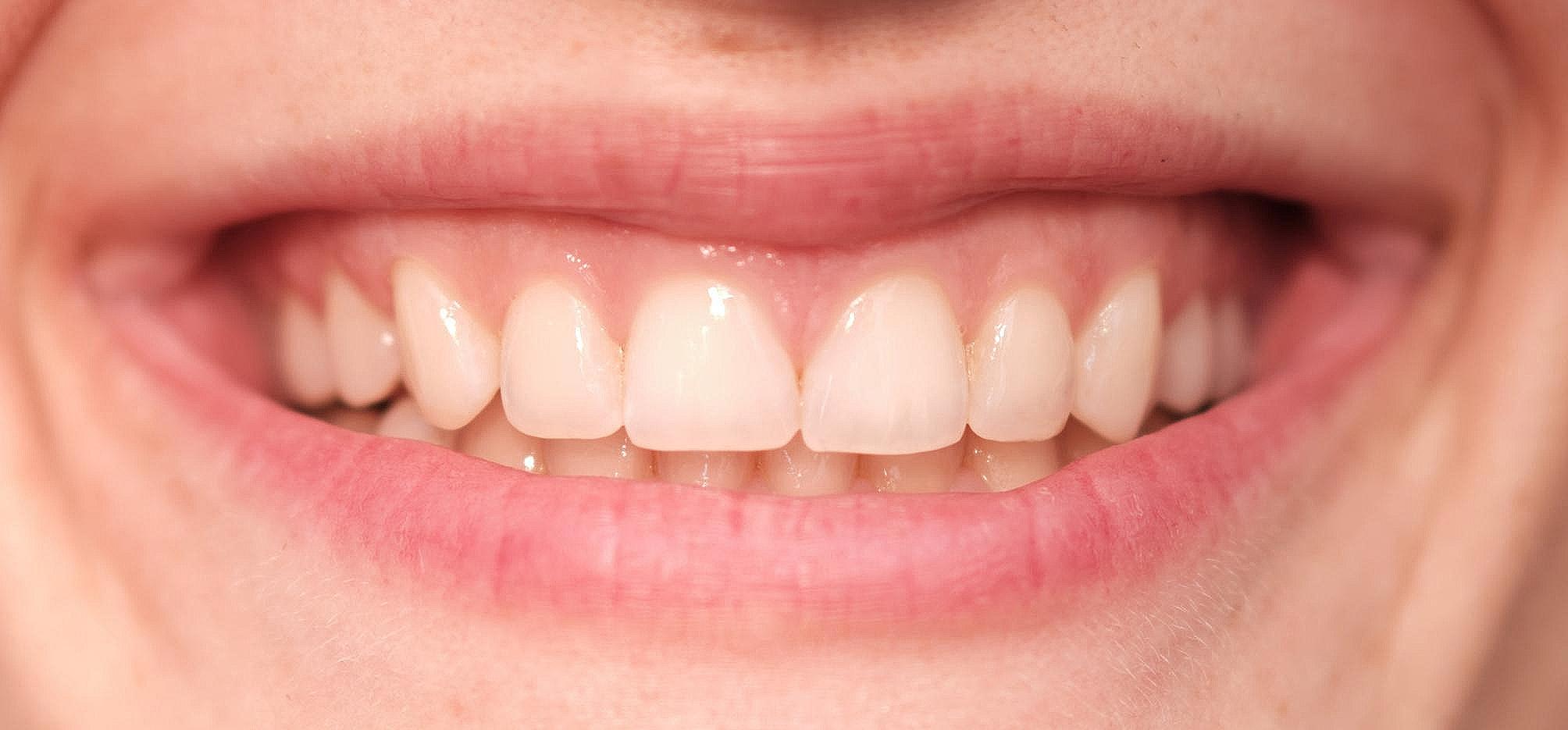
Reference photograph

A group of five specialists in Prosthodontics and Restorative dentistry and five laypersons scored (1–10; higher score better aesthetics) photographs of a smile of 20 persons without any dentoalveolar anomalies with visible anterior teeth, gingiva and lips. The photograph which received the highest-rated score was of a young female with A1 tooth shade according to the Vita color key and was chosen as a reference photograph (RP) (Fig. 1).

The photograph was taken under standardized conditions and camera settings while the model was placed next to a white wall in an upright position and with wide smile. The Fujifilm X-Pro 3 (Fujifilm, Minato City, Tokyo, Japan) camera with Fujinon XF50mmF2 R WR lens (Fujifilm, Minato City, Tokyo, Japan) was mounted on a tripod to ensure parallel position and the distance of 39 cm from the camera to the subject. A gray card was placed next to the model’s face, which served as a color calibrator in digital analysis (WhiBal, Michael Tapes Design, USA) [22]. The following settings have been set on the camera: ISO 100 sensor sensitivity, 1/125 shutter speed (SS), f2 aperture, 5500 K white balance (WB), and 10 MP resolution. The photograph was taken under standardized lighting conditions, in a room without a natural light source and with neon lighting (4 × 120 cm, 36 W, color 765, the light temperature of 5080 K, and illumination of 500 lx). Room temperature and lighting were measured with a Chroma-2 colorimeter (Lisun Electronics, Shanghai, China) [23]. Prior the photo was taken, the model’s teeth were professionally cleaned and polished (Proxyt RDA 83; Ivoclar Vivadent, Liechtenstein) and the tooth shade was measured using the pre-calibrated spectrophotometer (VITA Easyshade V, VITA Zahnfabrik, Bad Sackingen, Germany). The tip of the measuring probe was placed in the center of the middle third of the right maxillary central incisor. Measured CIE (Commission Internationale de l’Elcairage) L * a * b * values ​​(L = brightness, achromatic axis; a = chromatic axis red-green; b = chromatic axis yellow-blue) were recorded: L = 84.8, a = -2.2, b = 14.6.

### Digital manipulation of the reference photograph

**Fig. 2:**
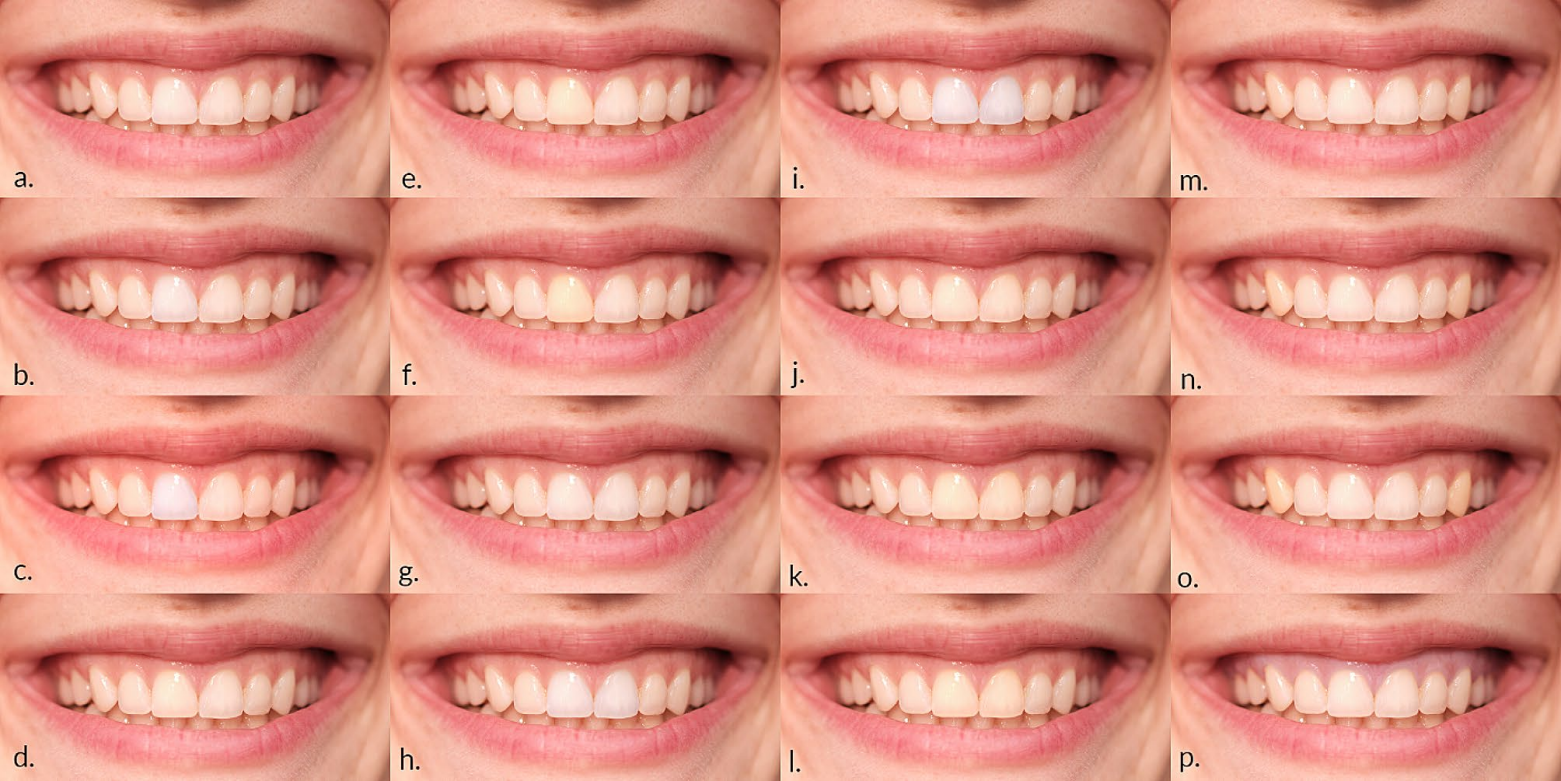
Tooth shade changes: **a.** lighter tooth shade change of the right maxillary central incisor to 5100 K; **b.** lighter tooth shade change of the right maxillary central incisor to 4700 K; **c.** lighter tooth shade change of the right maxillary central incisor to 4300 K; **d.** darker tooth shade change of the right maxillary central incisor to 5900 K; **e.** darker tooth shade change of the right maxillary central incisor to 6300 K; **f.** darker tooth shade change of the right maxillary central incisor to 6700 K; **g.** lighter tooth shade change of both maxillary central incisors’ to 5100 K; **h.** lighter tooth shade change of both maxillary central incisors’ to 4700 K; **i.** lighter tooth shade change of both maxillary central incisors’ to 4300 K; **j.** darker tooth shade change of both maxillary central incisors’ to 5900 K; **k.** darker tooth shade change of both maxillary central incisors’ to 6300 K; **l.** darker tooth shade change of both maxillary central incisors’ to 6700 K; **m.** darker tooth shade change of maxillary canines’ change to 5900 K; **n.** darker tooth shade change of maxillary canines’ change to 6300 K; **o.** darker tooth shade change of maxillary canines’ change to 6700 K; **p.** gingival color change to 4300 K

**Fig. 3:**
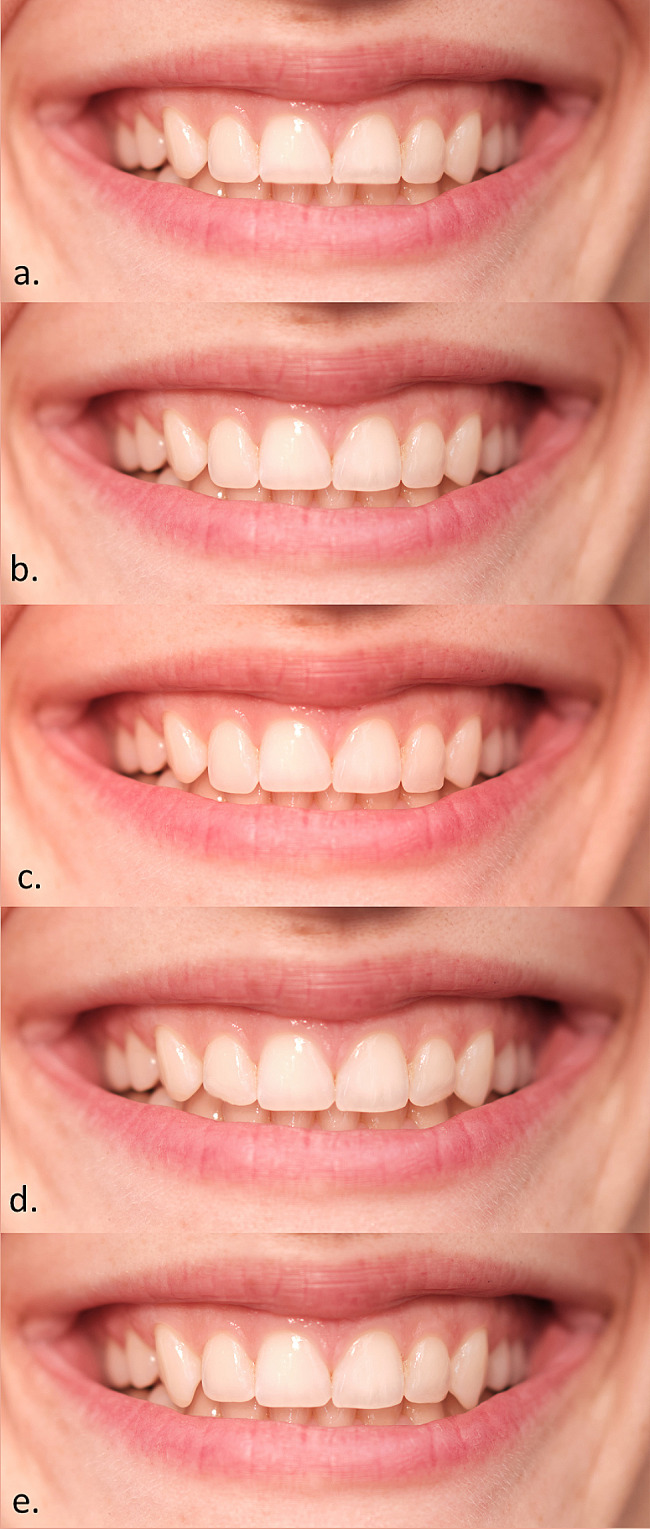
Length changes: **(a)** Shortened maxillary central incisors; **(b)** Elongated maxillary lateral incisors to the length of central incisors; **(c)** Elongated incisal edges of maxillary lateral incisors to the length of canines; **(d)** Beveled maxillary lateral incisors’ distal edge; **(e)** Elongation of both canines beyond the length of maxillary second incisors

**Fig. 4:**
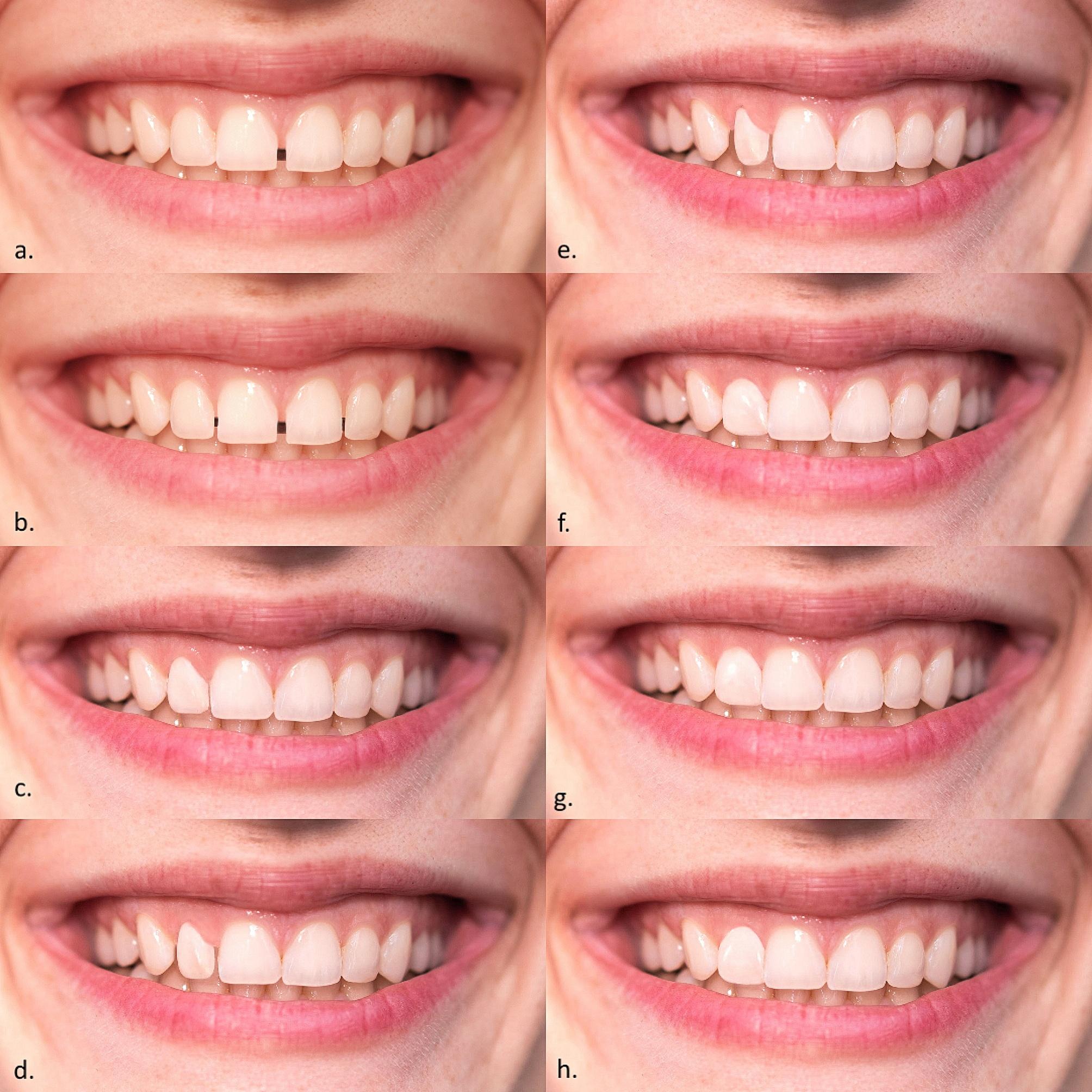
Position changes: **(a)** central diastema; **(b)** multiple diastemas **(c)** typical rotation of right maxillary lateral incisors + 10°; **(d)** typical rotation of right maxillary lateral incisors + 20°; **(e)** typical rotation of right maxillary lateral incisors + 30°; **(f)** atypical rotation of right maxillary lateral incisors − 10°; **(g)** atypical rotation of right maxillary lateral incisors − 20°; **(h)** atypical rotation of right maxillary lateral incisors − 30°

Photo manipulation of RP was performed using Adobe Photoshop (v.20.0.0. Adobe Inc., San Jose, California, USA). Manipulations included changes in tooth shade, length, and position of one or more maxillary teeth and gingiva (Figs. 2, 3 and 4). The white balance (WB) value for RP was 5500 K.

The shade change (Fig. 2) was achieved by gradually changing the WB, for the amount of 400 K, between each manipulated photograph (MP). Tooth shade changes included: (a) tooth shade change of the right maxillary central incisor; (b) tooth shade change of both maxillary central incisors, (c) tooth shade change of both maxillary canines, and (d) gingival color change. To obtain lighter tooth shades the WB was adjusted to 5100 K, 4700 K, and 4300 K, and to obtain the darker shades the WB was adjusted to 5900 K, 6300 K, and 6700 K for incisors. The shade manipulation of canines included only darker WB values ​​of 5900 K, 6300 K, and 6700 K, while gingival color change included only one manipulation of lighter color to 4300 K.

The length changes included: (a) shortened both maxillary central incisors (shortened cervical-incisal length); (b) elongated both maxillary lateral incisors to the length of central incisors, (c) elongated incisal edges of both maxillary lateral incisors to the length of canines, (d) beveled both maxillary lateral incisors distal edge, and (e) elongation of both canines beyond the length of maxillary first incisors (Fig. 3).

Photographs of altered tooth position included six photographs with the right maxillary lateral incisor rotations and photographs of central and multiple diastemas (Fig. 4). The right maxillary lateral incisor was chosen because rotations of the lateral incisors are relatively frequent and present an orthodontic problem. An intraoral scan (3Shape, Copenhagen, Denmark) of the RF teeth was done and the software simulated rotations with a deviation of 10 degrees from the initial situation, i.e. three typical (+ 10 + 20, +30 degrees) and three atypical (-10, -20, -30 degrees) rotations were made. For each rotation, the rotated maxillary lateral incisor was cut out and inserted within the RP using Adobe Photoshop. The use of intraoral scanner and its software to simulate tooth rotations is an original idea of the authors. A total of 29 manipulations were done.

### Online survey

The survey consisted of 37 questions divided into five sections. The first section incorporated a brief description of the research and written consent to participate in the research. The second contained general information about the participants (gender, age, year of study, presence of dental professionals in the immediate family, the experience of assistance) and the RP. The next three sections were thematically divided into tooth shade, length, and position modifications. Each of the tasks consisted of two photographs, the upper photograph was the RP, and the lower photograph was one of the MPs. In that way, comparisons between the RP and the MPs were enabled for each modification. The task was to evaluate the aesthetic acceptability of the RP and the MPs by attributing scores from 1 to 10 (1 indicated the least acceptable and 10 the most aesthetically pleasing smile).

### Statistical analysis

Using the IBM SPSS Statistics (v.20.0, Armonk, New York, USA) descriptive statistics (means and standard deviations), one-way Kolmogorov-Smirnov test, *X*^2^ test, 3-way MANOVA analysis (General Linear Model) and t-test were performed. The significance level was set at *p* < .05.

## Results

A total of 208 students from the School of Dental Medicine, University of Zagreb participated; 34 were the first, 42 the second, 37 the third, 37 the fourth, 30 the fifth, and 28 the sixth-year students. The whole sample included 175 (84.13%) females, and 33 (15.87%) males. According to the level of education, students were divided into the preclinical group (the first, second and third-year students, *n* = 113, 54%; 19 males) and clinical group (the fourth, fifth and sixth-year students, *n* = 95; 45.67%, 14 males). There was no significant difference in proportion of female and male students between the clinical and preclinical group (*X*^2^ = 0.17; *P* = .68). Furthermore, 104 students had the experience of assisting in dental office or had a dentist in the immediate family; 33 of them were preclinical (29.2%) and 71 were clinical students (74.7%). Significantly more clinical students had experience in assisting compared to preclinical students (*X*^2^ = 42.80; *p* < .001).

Descriptive statistics of the obtained scores for the RP and the MPs assessments depending on being the preclinical or clinical student, female or male, and the experience of assisting in dental office or having a dentist in the immediate family is presented in Table 1. The significance of the differences obtained by the 3-way MANOVA is presented in Table 2. Clinical students scored the RP and the MPs with lower scores than preclinical students and showed less dispersion in assessments (smaller standard deviations). No significant effect existed between the clinical and preclinical students for the RP, or for the small tooth shade change (+/- 400 K), while clinical students scored larger shade changes, especially those towards lighter modifications with significantly lower scores. Furthermore, clinical students’ scores for the MPs of the tooth length and position were also significantly lower than the preclinical students’ scores, except for the central diastema. The only significant difference between gender was that female students scored lighter tooth shade modifications with lower scores than males. Students who had experience in assisting or had a dentist in close family, mostly gave a bit lower score to the MPs than those who did not assist or had not a dentist in the family. However, without any statistical significance. Moreover, when only preclinical students were selected, t-test also showed no significant differences (*p* > .05) between those who assisted or had a dentist in the immediate family and those who did not. Combined interaction of gender and of being a clinical or preclinical student showed significant effect only for two-dimension modifications, such as elongated incisal edges of both lateral maxillary incisors beyond incisal edges of central incisors (F = 4.2, *p* < .05) and for shortened incisal edges of both maxillary incisors (F = 4.1, *p* < .05). Combined interaction of assisting and of being a clinical or preclinical student showed no significant effects, while combined interaction of gender and assisting showed significant effects (*p* < .05) only for some single tooth shade change (right maxillary incisor’s lighter shade at 4700 K, darker shade at 5900 and 6300 K).

**Table 1:** Descriptive statistics (x = mean values, SD = standard deviations) of assessments on the scale 1–10 (1-least acceptable; 10-the most pleasing esthetically) of Preclinical and Clinical students, Female and Male students, and students who had experience in assisting or had a dentist in the immediate family

					Preclinical students *n* = 113	Clinical students *n* = 95		Females *n* = 175	Males *n* = 175		Assisting or a dentist in close family *n* = 104	Not assisting or no dentists in close family *n* = 104
	**Photograph**				x (SD)	x (SD)		x (SD)	x (SD)		x (SD)	x (SD)
**T** **O** **O** **H** **S** **H** **A** **D** **E**	**Reference photograph** **(5500 K)**				**8.31 (1.41)**	**8.06 (1.30)**		**8.22 (1.39)**	**8.06 (1.22)**		**8.03 (1.50)**	**8.36 (1.19)**
	lighter	Right maxillary incisor at 5100 K			7.16 (1.71)	5.97 (1.75)		6.54 (1.85)	6.91 (1.65)		6.30 (1.73)	6.90 (1.97)
		Right maxillary incisor at 4700 K			5.28 (2.35)	3.83 (1.62)		4.49 (2.12)	5.18 (2.30)		4.20 (1.93)	4.99 (2.31)
		Right maxillary incisor at 4300 K			4.05 (2.31)	2.57 (1.80)		3.22 (2.13)	4.06 (2.47)		2.91 (1.92)	3.97 (2.39)
	darker	Right maxillary incisor at 5900 K			7.41 (1.37)	6.72 (1.84)		7.07 (1.62)	7.18 (1.79)		7.03 (1.79)	7.14 (1.40)
		Right maxillary incisor at 6300 K			6.01 (1.79)	5.02 (1.87)		5.49 (1.87)	5.85 (2.00)		5.49 (1.83)	5.60 (1.96)
		Right maxillary incisor at 6700 K			4.36 (2.07)	3.38 (1.80)		3.82 (1.98)	4.30 (2.07)		3.63 (1.85)	4.16 (2.11)
	lighter	Both maxillary incisors at 5100 K			6.32 (2.04)	5.55 (1.95)		5.88 (2.03)	6.33 (2.03)		5.79 (2.12)	6.13 (1.93)
		Both maxillary incisors at 4700 K			4.14 (2.18)	3.06 (1.62)		3.52 (1.96)	4.33 (2.27)		3.37 (1.84)	3.93 (2.17)
		Both maxillary incisors at 4300 K			3.35 (2.18)	2.07 (1.37)		2.61 (1.82)	3.64 (2.46)		2.43 (1.74)	3.11 (2.12)
	darker	Both maxillary incisors at 5900 K			7.55 (1.75)	7.49 (1.49)		7.50 (1.74)	7.61 (1.22)		7.63 (1.68)	7.41 (1.65)
		Both maxillary incisors at 6300 K			5.90 (1.94)	5.62 (1.93)		5.74 (1.88)	5.94 (2.16)		5.72 (2.00)	5,83 (1.86)
		Both maxillary incisors at 6700 K			5.56 (2.07)	5.32 (2.01)		5.43 (2.10)	5.52 (2.05)		5.39 (2.07)	5.50 (2.12)
	darker	Both maxillary canines at 5900 K			7.54 (1.71)	7.51 (1.58)		7.54 (1.69)	7.42 (1.41)		7.50 (1.71)	7.55 (1.58)
		Both maxillary canines at 6300 K			6.53 (1.99)	6.51 (1.90)		6.58 (1.97)	6.48 (1.75)		6.65 (1.97)	6.48 (1.99)
		Both maxillary canines at 6700 K			5.09 (2.23)	4.89 (2.13)		4.99 (2.25)	5.00 (2.14)		4.97 (2.17)	5.02 (2.29)
	Lighter gingiva (4300 K)				5.66 (2.73)	5.47 (2.71)		5.77 (2,67)	4.55 (2.76)		5.69 (2.73)	5.45 (2.71)
**L** **E** **N** **G** **T** **H**	Shortened both maxillary central incisors				5.07 (2.17)	4.74 (2.11)		4.94 (2.09)	4.79 (2.45)		4.71 (2.16)	5.07 (2.14)
	Elongated both maxillary lateral incisors to the length of central incisors				7.01 (2.18)	5.84 (2.02)		6.49 (2.12)	6.39 (2.44)		6.17 (2.12)	6.78 (2.18)
	Elongated incisal edges of both maxillary lateral incisors to the length of canines				5.96 (2.40)	5.25 (2.10)		5.63 (2.29)	5.7 (2.62)		5.38 (2.40)	5.89 (2.25)
	Beveled both maxillary lateral incisors distal edge				5.97 (2.46)	4.55 (2.45)		5.27 (2.46)	5.45 (3.01)		4.84 (2.41)	5.77 (2.61)
	Elongated canines				5.58 (2.69)	4.62 (2.52)		5.15 (2.61)	5.09 (2.92)		4.89 (2.68)	5.39 (2.62)
**P** **O** **S** **I** **T** **I** **O** **N**	Central diastema				4.60 (2.41)	4.15 (2.10)		4.45 (2.32)	4.12 (2.12)		4.25 (2.31)	4.54 (2.28)
	Multiple diastemas				5.02 (0.30)	4.98 (0.20)		5.00 (0.29)	5.03 (0.25)		4.98 (0.34)	5.02 (0.17)
	Typical rotation of the right lateral maxillary incisor		+ 10^0^		6.58 (2.20)	5.67 (1.98)		6.23 (2.22)	5.82 (2.11)		5.91 (2.13)	6.42 (2.25)
			+ 20^0^		4.23 (2.39)	3.52 (1.79)		3.96 (2.13)	3.61 (2.30)		3.65 (1.90)	4.15 (2.37)
			+ 30^0^		3.13 (2.05)	2.56 (1.54)		2.92 (1.83)	2.61 (1.95)		2.71 (1.69)	3.03 (2.00)
	Atypical rotation of the right lateral maxillary incisor		− 10^0^		5.99 (2.27)	5.04 (2.17)		5.61 (2.54)	4.93 (2.68)		5.28 (2.40)	5.84 (2.11)
			− 20^0^		6.20 (2.31)	4.95 (2.11)		5.73 (2.24)	5.09 (2.57)		5.31 (2.35)	5.95 (2.22)
			− 30^0^		5.50 (2.56)	3.82 (2.17)		4.81 (2.51)	4.36 (2.61)		4.37 (2.51)	5.11 (2.50)

**Table 2:** Three-way multiple ANOVA results of the effects of gender, experience in assisting or having a dentist in immediate family or not, and of belonging to the clinical or preclinical group

							Corrected Model				Effect of being Preclinical or Clinical Student				Effect of Gender				Effect of Assisting or having a Dentist as a Close Family Member or not		
	**Photograph**						df	*F*	*p*		df	*F*	*P*		df	*F*	*p*		df	*F*	*p*
**T** **O** **O** **H** **S** **H** **A** **D** **E**		**Reference photograph** **(5500 K)**					**7**	**0.929**	**0.485 N.S.**		**1**	**0.351**	**0.554 N.S.**		**1**	**0.202**	**0.654 N.S.**		**1**	**0.922**	**0.338 N.S.**
			Right maxillary incisor at 5100 K				7	4.797	< 0.001**		1	2.580	0.110 N.S.		1	1.724	0.191		1	0.922	0.338 N.S.
		lighter	Right maxillary incisor at 4700 K				7	5.576	< 0.001**		1	5.609	0.01 *		1	4.910	0.028*		1	1.567	0.212 N.S.
			Right maxillary incisor at 4300 K				7	5.493	< 0.001**		1	4.356	0.038*		1	7.339	0.007**		1	3.134	0.078 N.S.
		darker	Right maxillary incisor at 5900 K				7	2.972	0.005**		1	3.047	0.082 N.S.		1	0.261	0.610 N.S.		1	3.819	0.055 N.S.
			Right maxillary incisor at 6300 K				7	4.183	< 0.001**		1	3.615	0.059 N.S.		1	1.841	0.176 N.S.		1	0.461	0.498 N.S.
			Right maxillary incisor at 6700 K				7	3.719	0.001**		1	6.766	0.010*		1	2.055	0.153 N.S.		1	0.371	0.543 N.S.
		lighter	Both maxillary incisors at 5100 K				7	1.810	0.087 N.S.		1	4.076	0.045*		1	0.472	0.493 N.S.		1	1.115	0.292 N.S.
			Both maxillary incisors at 4700 K				7	4.524	< 0.001**		1	8.219	0.005**		1	3.648	0.058 N.S.		1	0.133	0.715 N.S.
			Both maxillary incisors at 4300 K				7	6.398	< 0.001**		1	12.022	0.001**		1	6.709	0.010*		1	1.405	0.237 N.S.
		darker	Both maxillary incisors at 5900 K				7	1.143	0.338 N.S.		1	0.330	0.567 N.S.		1	0.042	0.838 N.S		1	2.357	0.126 N.S.
			Both maxillary incisors at 6300 K				7	1.634	0.128 N.S.		1	0.040	0.841 N.S.		1	1.310	0.254 N.S.		1	0.281	0.597 N.S.
			Both maxillary incisors at 6700 K				7	1.360	0.224 N.S.		1	0.277	0.599 N.S.		1	0.302	0.583 N.S.		1	0.525	0.470 N.S.
			Both maxillary canines at 5900 K				7	0.623	0.737 N.S.		1	0.019	0.890 N.S.		1	0.182	0.671 N.S.		1	0.390	0.533 N.S.
		darker	Both maxillary canines at 6300 K				7	0.888	0.517 N.S.		1	0.096	0.758 N.S.		1	0.196	0.658 N.S.		1	0.043	0.836 N.S.
			Both maxillary canines at 6700 K				7	0.839	0.556 N.S.		1	1.780	0.184 N.S.		1	0.353	0.553 N.S.		1	0.239	0.626 N.S.
	Lighter gingiva (4300 K)						7	1.991	0.058 N.S.		1	0.688	0.408 N.S.		1	1.988	0.160 N.S.		1	0.219	0.641 N.S.
**L** **E** **N** **G** **T** **H**	Shortened both maxillary central incisors						7	1.522	0.162 N.S.		1	4.518	0.035*		1	0.944	0.332 N.S.		1	0.309	0.579 N.S.
	Elongated both maxillary lateral incisors to the length of central incisors						7	3.523	0.001**		1	11.030	0.001**		1	0.439	0.508 N.S.		1	0.389	0.533 N.S.
	Elongated incisal edges of both maxillary lateral incisors to the length of canines						7	1.687	0.114 N.S.		1	6.357	0.012*		1	0.128	0.721 N.S.		1	0.269	0.604 N.S.
	Beveled both maxillary lateral incisors distal edge						7	4.490	< 0.001**		1	13.935	< 0.001**		1	0.011	0.918 N.S.		1	1.481	0.225 N.S.
	Elongated canines						7	2.965	0.006**		1	8.926	0.003**		1	0.074	0.786 N.S.		1	0.943	0.333 N.S.
**P** **O** **S** **I** **T** **I** **O** **N**	Central diastema						7	0.740	0.638 N.S.		1	1.835	0.177 N.S.		1	1.609	0.206 N.S.		1	0.488	0.485 N.S.
	Multiple diastemas						7	0.61	0.740 N.S.		1	0.112	0.739 N.S.		1	0.189	0.664 N.S.		1	0.022	0.882 N.S.
	Typical rotation of the right lateral maxillary incisor				+ 10^0^		7	2.389	0.023*		1	9.606	0.002**		1	2.546	0.112 N.S.		1	0.140	0.709 N.S.
					+ 20^0^		7	1.738	0.102 N.S.		1	6.092	0.014*		1	1.277	0.260 N.S.		1	0.031	0.861 N.S.
					+ 30^0^		7	1.357	0.226 N.S.		1	4.899	0.028*		1	1.308	0.254 N.S.		1	0.070	0.792 N.S.
	Atypical rotation of the right lateral maxillary incisor				− 10^0^		7	2.548	0.016*		1	7.259	0.008**		1	3.602	0.053 N.S.		1	0.091	0.763 N.S.
					− 20^0^		7	2.970	0.006**		1	10.087	0.002*		1	3.004	0.085 N.S.		1	0.065	0.798 N.S.
					− 30^0^		7	4.406	< 0.001**		1	16.276	< 0.001**		1	1.887	0.171 N.S.		1	0.190	0.663 N.S.

df = Degree of Freedom; F = *F* value, *P* = probability; *=*p* < .05; **=*p* < .01; N.S.=not significant

## Discussion

Numerous studies have proven divergence in the perception of aesthetically appealing smiles, with laypeople accepting a wider range of deviations from common aesthetic norms than dental professionals [11, 24–30].

The objective of this study was to study whether there is a difference in the perception of tooth shade change and anatomical variations of anterior maxillary teeth among dental students depending on the level of education, gender, experience in assisting in a dental office and the presence of a dentist in the immediate family. The research was conducted among preclinical and clinical students evaluating the aesthetic acceptability of a smile on the unaltered, reference photograph (RP) and on the other 28 modified photographs (MP).

The results of the present study proved that preclinical students did not notice small modifications or accepted a wider range of tooth shade, length, and position modifications in comparison to clinical students. The knowledge of preclinical students is scarce, they have not had the opportunity to practice their perception in preclinical education. However, the “ideal” (RP) was best rated in both groups without significant difference. Omar and Tai [27] also showed that dental students rated the ideal photograph with the highest score, and significantly lower photographs with esthetic deviations. Obviously, targeted clinical education influenced clinical students in the present study to notice changes earlier and/or adopt more critical aesthetic norms. Similar conclusion was made by Mannaa [28], stating that dental students’ exposure and awareness of esthetic dentistry topics increased with academic progression.

This study also showed that MPs with the highest deviations towards lighter spectrum in the category of tooth shade were rated with the lowest scores, especially in clinical students. Relatively high scores were assigned to photographs showing small deviations in the category of tooth shade change towards darker spectrum (WB 5900 K), without significant difference between clinical and preclinical students, which can be attributed to the natural, yellowish shade of the teeth, especially canines, which are slightly darker in natural dentition. However, larger modifications towards darker spectrum were scored worse in both groups. Some studies proved that color-matching skills can be improved by education, knowledge [31–34] and targeted color matching training [35], which is in line with our results, especially for the perception of lighter shades of clinical students. Practice and education sharpen students’ clinical ability to recognize different tooth shades and match shades with restorative and prosthetic materials. Although clinical students gave lower scores to all MPs than preclinical students, the lowest scores in both groups were given to the lightest shades and the midline diastema. Noureddine et al. [36] examined multiple diastemas of anterior teeth and midline diastema and found out that the midline diastema was rated worst, suggesting that negative impact was related to the width of a midline diastema. In similar study clinical students ranked a midline diastema to be significantly lower than preclinical students who perceived the smile with retroclined incisors as significantly less pleasing than the clinical students. Together with midline diastema, open bite was perceived as an unfavorable feature by both year-groups [37].

A significant difference in the evaluation of tooth rotations between clinical and preclinical students could not be compared with other studies as no documented studies were found in the available dental literature. Alhammadi et al. [38] examined the perception of different aesthetic parameters of the orofacial region in students of the preclinical and clinical groups and found that clinical students were more successful in recognizing minimal deviations as opposed to preclinical students, which is in line with our results. Khalaf et al. [39] also showed that the higher the level of dental education (dental specialists, dentists, dental students, and laypersons), the lower the aesthetic perception and that dental educational level influences the aesthetic perception of anterior crowding and spacing, which may have an impact on treatment planning and need for orthodontic intervention. Although they have proven that education (students vs. dentists) has an impact on aesthetic perception, they did not make a detailed analysis between students of different years, and only clinical (fourth, fifth and sixth year) were included.

Professional knowledge can, besides targeted academic education, also be obtained from other sources such as assisting in a dental office or from a presence of a dentist in the immediate family [40, 41]. Therefore, this study also aimed to examine students’ knowledge acquired by assisting or from a dentist – a close family member. No significant effect of assisting or having a dentist in the immediate family on perception of color, shape and tooth position was observed (*p* > .05). Furthermore, as clinical students who reported having experience in assisting acquired knowledge also in academic education, only preclinical students were selected and there was no significant difference between those who assisted or did not (t-test, *p* > .05). Obviously, targeted academic education was more efficient in acquiring knowledge and skills required to notice deviations and modifications related to teeth shade, dimensions and positions, which was confirmed by significantly lower scores of clinical students in this study.

The only significant effect of gender was that females gave significantly lower scores for MPs towards lighter spectrum than male students, while the perception of dimension or position modifications showed no significant effect. In similar studies that dealt with the difference in the perception of aesthetic deviations, a greater criticism of female participants was also proven [42]. Color recognition difficulties are more common in men than in women [43, 44]. In investigating the tolerance thresholds of aesthetic deviations, the results of previous studies are contradictory. While some emphasize the important role of practice and experience in accurately determining and thus perceiving color [45–48], others argue that experience has minimal impact on the ability to accurately determine tooth shade [49–51].

It is important to discuss certain limitations of this study. To ensure good color vision the exclusion criteria was set at the error score > 20 (FM hue test). However, it allowed some small differences in color vision between gender. Some other factors like influence from the social media, fashion changes, etc. have not been included in this research. Studies on the aesthetics of smiles differ greatly depending on how data are collected (online surveys, self-smile evaluation, photographs, software photo manipulation), choice of parameters for evaluation, and evaluators’ subjective esthetics perception, making it difficult to compare results [42]. This study arbitrarily selected the characteristics of a smile that the authors considered important in the perception of a smile as aesthetically appealing. Other limitations of the present study are mood swings, tiredness, lack of motivation, and different type of PC screens during assessments.

The strength of the research is including the tooth rotations in smile aesthetic evaluation (which has not been included in previous studies), a sufficient number of participants in the preclinical and clinical groups, an equal ratio of female and male students similar to the proportion of gender distribution in the respective dental school, and evaluation of tooth shade, length, and tooth position modifications, analyzing mutual effects of belonging to the clinical or preclinical group, gender and possible knowledge acquired from other sources. One of the biggest strengths of the study is the use of the intraoral scanner and its software to simulate tooth rotations on a 2D photograph, which is novelty, and it has not been reported yet in the dental literature.

It is important to monitor students’ professional development and compare the impact of education on the criticism towards deviations from aesthetic norms. Understanding students’ perception and level of criticism depending on their level of education are of great importance because educators can get insight into the progression of their education. Senior students should be able to make critical clinical decisions considering the aesthetic components of a smile.

## Conclusions

Clinical students gave lower scores and were more consistent in the assessments of modifications of the pleasing smile of the reference photograph, especially for changes towards lighter spectrum, and changes in tooth dimensions and positions. Knowledge acquired through targeted academic education showed significant effect and sharpened the ability of clinical students to better perceive tooth shade, dimensions, and position changes, while knowledge acquired by assisting in dental office or having a dentist in the immediate family showed no significant effect.

## Data Availability

The datasets used and/or analysed during the current study available from the corresponding author on reasonable reques.
